# Investigating the point prevalence, types, severity, causes and predictors of vaccines administration errors during COVID-19 pandemic in Jordan

**DOI:** 10.1371/journal.pone.0312050

**Published:** 2025-01-03

**Authors:** Derar H. Abdel-Qader, Hasan Abdel-Qader, Jennifer Silverthorne, Chuenjid Kongkaew, Moh’d Al Nimrawi, Ahmad Z. Al Meslamani, Nathir M. Obeidat, Wail Hayajneh, Feras Hawari, Souraya Z. Arabi, Salahdein AbuRuz

**Affiliations:** 1 Faculty of Pharmacy & Medical Sciences, University of Petra, Amman, Jordan; 2 Al Rashid Hospital Center, Amman, Jordan; 3 Ministry of Health, Amman, Jordan; 4 Division of Pharmacy & Optometry, The University of Manchester, Manchester, United Kingdom; 5 Department of Pharmacy Practice, Naresuan University, Phitsanulok, Thailand; 6 International Group for Educational Consultancy (IGEC), Sydney, Australia; 7 AAU Health and Biomedical Research Center, Al Ain University, Abu Dhabi, United Arab Emirates; 8 College of Pharmacy, Al Ain University, Abu Dhabi, United Arab Emirates; 9 School of Medicine, The University of Jordan, Amman, Jordan; 10 School of Medicine, St. Louis University, St. Louis, MO, United States of America; 11 School of Medicine, Jordan University of Science & Technology, Irbid, Jordan; 12 School of Medicine, Beirut Arab University, Beirut, Lebanon; 13 Department of Pharmacology and Therapeutics, College of Medicine and Health Sciences, The United Arab Emirates University, Al Ain, United Arab Emirates; Universite Ziane Achour de Djelfa Faculte des Sciences de la Nature et de la Vie, ALGERIA

## Abstract

**Background:**

There is a paucity of research regarding COVID-19 vaccines administration errors (VAEs) during the COVID-19 pandemic. This study aimed to investigate the prevalence, types, severity, causes and predictors of VAEs in Jordan during the recent pandemic.

**Method:**

This was a 3-day (Sunday, Tuesday and Thursday of the third week of November 2021) prospective, covert observational point prevalence study. It involved direct observation of vaccination administration practices by covert observers who recorded data on a standardized form, documenting the administration process, observed errors, and contextual factors, such as workload, distractions, and interruptions directly after each observation. Univariate and multivariable logistic models were constructed in order to identify predictors of VAEs.

**Results:**

The point prevalence of VAEs was 2.4% (209 errors / 8743 vaccine doses). These VAEs were categorized into six types: timing (interval) error (69, 33.0%) dosing error (60, 28.7%), incorrect vaccine product (42, 20.1%), site/route error (17, 8.1%), documentation error (15, 7.2%), and other (6, 2.9%). Most errors were minor (133, 63.6%) and moderate (63, 30.1%). There were 174 (54.9%) healthcare provider-related contributing factors and 102 (32.2%) patient-related factors. Receiving the vaccine in the Southern region compared to Capital region (aOR: 1.92; 95% confidence intervals, 95%CI: 1.41–2.49; p = 0.001) and receiving the vaccine during peak hours compared to regular hours (aOR: 2.18; 95%CI: 1.58–3.86; p = 0.002) were significant predictors of VAEs.

**Conclusion:**

Though infrequent, VAEs had prevalence higher than previously reported in the literature, posing serious public health challenges, which might have compromised immunization efficacy and patient safety. Identifying these errors’ causes and formulating strategies to reduce them is crucial for enhancing vaccination results.

## Introduction

Vaccination stands as one of the most impactful public health interventions in history, effectively curbing the spread of infectious diseases and saving countless lives [1–3]. The COVID-19 pandemic underscored the critical role of vaccination in global health, prompting an unprecedented effort to develop, distribute, and administer vaccines at a global scale [4]. Jordan, in response to the pandemic, implemented a phased vaccination programme beginning in December 2020, prioritizing high-risk groups and aiming for widespread immunization coverage [5]. By September 2022, over 10.5 million doses of four different COVID-19 vaccines (Pfizer-BioNTech, Sinopharm, Sputnik V, and Oxford-AstraZeneca) had been administered in the country [6].

While vaccination is generally safe and effective, errors during the administration process can occur, potentially jeopardizing this essential public health tool. Vaccine administration errors (VAEs) encompass a range of preventable incidents that deviate from established guidelines and best practices, ranging from incorrect vaccine selection or dosage to improper storage, handling, and administration techniques [7, 8]. Such errors not only impact individual patient safety [9–11], but can also diminish vaccine efficacy, leading to inadequate protection against targeted pathogens [12]. Furthermore, VAEs can erode public trust in vaccination programmes, potentially fueling vaccine hesitancy and hindering efforts to achieve herd immunity [13]. Understanding the causes and contributing factors of VAEs is crucial for developing targeted interventions [14, 15].

Prior to the COVID-19 pandemic, research on VAEs highlighted a concerning prevalence, with estimates ranging from 0.005 to 141.69 errors per 10,000 doses administered [16]. The most frequently reported errors included administering the wrong vaccine, off-schedule vaccination, and dosage errors [17]. However, the unprecedented scale and urgency of the COVID-19 vaccination campaign, coupled with the introduction of novel vaccines with unique storage and administration requirements, raised concerns about a potential surge in VAEs [4, 18].

Despite the potential for increased errors during the COVID-19 vaccination effort, there remained a significant gap in research specifically examining the prevalence, types, and causes of VAEs related to COVID-19 vaccines, particularly within the Jordanian context. Understanding the frequency and nature of these errors during this crucial public health initiative is essential for informing targeted interventions, optimizing vaccination safety, and maximizing the impact of vaccination programmes. Therefore, this study aimed to investigate the point prevalence, types, severity, causes, and predictors of COVID-19 VAEs during the initial phase of the mass vaccination campaign in Jordan.

## Methods

### Study design

This was a prospective observational point prevalence study carried out to address the prevalence, types, severity, and predictors of VAEs. Additionally, researchers interviewed vaccinators to investigate the factors contributing to these VAEs.

### Operational definitions

- Vaccine administration errors (VAEs) refer to any preventable event that could result in the inappropriate use of a vaccine or harm to the individual receiving the vaccine.
- Covert observers: members of the research team selected to observe the vaccination process and investigate VAEs.
- Vaccinators: people who were giving vaccines.
- Vaccinees: people who were receiving vaccines.
- Point prevalence of administration errors is a measure of the proportion of administration errors in a vaccination campaign at a particular time.

The different types and severity of errors were defined and classified based on the definitions and classifications provided by the Centers for Disease Control and Prevention [17]. The used VAE audit form included types and severity of errors adapted and validated by a multidisciplinary committee, consisting of three experts in infectious disease pharmacotherapy, public health, and nursing.

VAE severity and impact was considered, as follows: Minor VAEs, such as breaches in aseptic technique or minor deviations from recommended sites or schedules, are unlikely to cause substantial harm or notably alter vaccine efficacy. Moderate VAEs, such as incorrect dosage (unless grossly miscalculated), or misuse of the administration route, can reduce vaccine effectiveness and increase the risk of adverse events. Severe VAEs, as defined by the committee, include administering an incorrect vaccine or giving one to an individual with known severe allergies, potentially leading to harm and/or significantly diminishing the effectiveness of the vaccination. Life-threatening VAEs, although relatively rare, can lead to severe health complications or even cause death. These could include improper administration of vaccines intended for intramuscular use via intravenous route, or administering a vaccine to someone known to have severe allergic reactions to it.

To investigate the VAE contributing factors, we devised a detailed checklist, which categorized causes into three broad groups: healthcare provider-related factors, system-related factors, and patient-related factors. Healthcare provider-related factors included distractions, knowledge gaps (although trained, trainees have got gaps in proper vaccination procedure), heavy workloads, inadequate training (trainees have not attended the full training sessions), and miscommunication with other providers. System-related factors encompassed non-standardized administration protocols, low staffing levels, inefficient health information systems, and challenges with vaccine supply chain management. Patient-related factors included inadequate understanding of vaccination schedules, failure to disclose pertinent medical information, language barriers, and anxiety or fear of needles. This comprehensive checklist guided the interviews with vaccinators, ensuring an organized approach in collecting and categorizing data.

### Sampling methodology and sample size calculation

To investigate VAEs, a three-day (Sunday, Tuesday and Thursday) prospective observational study, during the third week of November 2021, was conducted in 10% (n = 11 centers) of all vaccination centers (**Table 1**), purposively selected to cover the largest centers in the central, northern and southern areas in Jordan. Vaccination centers had several stations; each station had vaccination team comprising of 3 to 4 vaccinators, who volunteered to administer vaccines during COVID-19 vaccination campaign in Jordan. Fifteen covert observers, of pharmacy, nursing and medical background, were purposively chosen from the vaccinators. The most experienced persons among the vaccinators were chosen to act as covert observers.

**Table 1:** COVID-19 vaccination centers in Jordan—the largest centers were considered each time.

Governorate	Total number of immunization centers	Number of included centers
Irbid	17	1
Balqa	9	0
Zarqa	10	1
Tafila	5	1
Capital	25	4
Aqaba	4	1
Karak	10	1
Mafraq	8	0
Jerash	6	0
Ajloun	5	1
Madaba	3	0
Maan	5	1
**TOTAL**	**107**	11

All vaccinators had to receive training in administering COVID-19 vaccines conducted by the Ministry of Health. This training focused on providing a standardized vaccination procedure regarding proper handling, storage, dilution, and correct administration procedure for the different COVID-19 vaccines. Covert observers received additional training on this observational study in particular, to ensure understanding of the standardized vaccination procedure and the proper conduct of the observational process for data collection.

All vaccinees presenting to the selected vaccination centers during the study period were eligible for inclusion. Vaccinees were excluded if they: (a) were unable to provide informed consent, (b) had documented severe allergies to any vaccine component, (c) presented with a significant acute illness on the day of vaccination, (d) had incomplete observation of the vaccine administration process, or (e) had crucial missing information in the National COVID-19 Vaccines Pharmacovigilance Registry.

To calculate the sample size of vaccine doses required, we used the highest prevalence of VAEs from the literature, which was 141.69 per 10,000 vaccine doses [16] to power the study. The highest point prevalence when determining sample size improves the reliability and validity of study findings [19]. The estimated sample size for accurately estimating the VAEs rate with a confidence level of 95% and a margin of error of 2% was approximately 135 vaccine doses. However, given the public health emergency of COVID-19 pandemic warranting massive vaccine doses, by including all cases, we eventually had many more than needed.

### Study setting and procedure

This study involved direct observation of vaccination administration practices by covert observers, who were part of the vaccination team. Covert observers were trained to observe closely the entire vaccination process completed by their colleagues in the vaccination station, and consequently complete a VAE audit form. The observations took place during scheduled vaccination sessions in selected centers over three days (of the third week of November 2021. Vaccination centers worked from Saturday to Thursday (8:00 AM to 3:00 PM).

To help remember the entire incident, covert observers recorded data on a standardized form, documenting the administration process, observed errors, and contextual factors, such as workload, distractions, and interruptions directly after each observation. Filling each form required few minutes to be completed. To enhance the completeness of data, all errors voluntarily reported by vaccinators were also recorded by observers. At the end of each data collection session, the multidisciplinary committee met to confirm and classify the types of errors, resolving any discrepancies through consensus discussions with a fourth public health expert.

The principal investigator piloted the observational process and audit form for three days to ensure accuracy and practicality of the entire procedure. To capture accurate information, semi-structured interviews with vaccinators committing errors took place immediately after the observation sessions on all days had finished.

To gain accurate information, the research team was granted full access to the National COVID-19 Vaccine Pharmacovigilance Registry. This access allowed them to view detailed information, such as the vaccinees’ gender, age, comorbidities, location (governorates), the type of vaccine administered, and the number of vaccine doses received. Not all of this information was used for the consequent predictor analysis.

### Data analysis

We exported the collected data to a Microsoft Excel spreadsheet (Microsoft Corporation, Redmond, WA, USA). We then cleaned and preprocessed the data collected from both the observation forms and interview sheets. This involved addressing missing data, ensuring consistency in categorization, and preparing the data for analysis As follows:

1. Observation Forms:
  ○ Example 1 (Missing Data): Some forms had missing entries (e.g., patient age not recorded). The researchers employed techniques like imputation (replacing missing values with estimated ones based on other data).
  ○ Example 2 (Data Inconsistency): Discrepancies in recorded data points existed (e.g., a single vaccine dose recorded as both "Pfizer" and "BNT162b2"). This required standardization to ensure consistent categorization.
2. Interview Sheets:
  ○ Example 1 (Categorization): The textual data from interviews needed categorization. For example, if a vaccinator attributed an error to "being busy," this response would need to be categorized under "heavy workload" or "distractions" based on the predefined list of contributing factors.
  ○ Example 2 (Combining Data): The categorized information from the interviews was then linked to the corresponding observation data, possibly creating new variables or adding depth to existing ones.

We used SPSS v.26 (IBM Corporation, Armonk, NY, USA) to enter the data for statistical analysis. In order to identify predictors of VAEs, we constructed univariate and multivariable logistic model. Potential predictors of VAEs used in univariate were age, gender, location, previous COVID-19 infection, time of vaccinations, vaccinated against seasonal influenza virus and type of vaccine. The occurrence of VAEs served as the dependent variable, and the vaccinators’ independent factors included: vaccination timing (peak hours [9:00–10:00 AM; 12:00–1:00 PM] vs. regular hours (other hours [11:00 AM– 12:00 PM; 2:00–3:00 PM])), gender (male as a reference), and age (>65 as a reference). Additionally, we considered vaccinees factors, such as vaccine type (BBIBP-CorV as a reference). Categorical variables were presented as counts with proportions, while continuous variables were expressed as means with standard deviation. We calculated and displayed 95% confidence intervals (95%CI) for adjusted odds ratios (aORs). Logistic regression was performed using the reverse Wald method with an entry set at 0.05 and removal at 0.1. Collinearity was checked using the variance inflation factor and tolerance. Statistical significance was determined by a p value of less than 0.05.

The information obtained from the semi-structured interviews was thoroughly reviewed and categorized based on the identified causes of VAEs. We ensured the anonymity of the interviewee’s identities throughout the categorization process.

### Ethical considerations

Research Ethics approval was obtained from the institutional review board from the Ministry of Health (REC-MOH-7722), for this study which was part of a large governmental programme generally pertaining to the safety and effectiveness of four types of COVID-19 vaccines. Covert observers were provided with detailed information about the study objectives, procedures, and potential risks or benefits before obtaining their written informed consent. Confidentiality and anonymity of vaccinators and vaccinees were strictly maintained throughout the study, and data were stored securely to ensure privacy and data protection. To minimize Hawthorne effect during the vaccination process, observers were covert to the vaccinators. If noticed by observers, any error, particularly those potentially reducing the effectiveness and safety of the vaccine, was intercepted to avoid causing harm to vaccinees.

## Results

The total number of doses of vaccinees included in this study was 8743, of which 2131 (24.4%) aged between 35 and 65 years, 4962 (56.8%) were females, and 3548 (40.6%) were immunized in the Capital region. Among the vaccinees, 2176 (24.9%) received the vaccine during peak hours. The most common vaccines given during the study were BNT162b2 (4511, 51.6%) and BBIBP-CorV (2681, 30.7%) where it was one per included vaccinee. (**Table 2)**. The point prevalence of VAEs was 2.4% (209 errors / 8743 vaccine doses).

**Table 2:** Characteristics of vaccinees (N = 8743).

Parameter		Total, n (%)
**Age (years**)	16–18	1863 (21.3%)
	18–35	3886 (44.4%)
	35–65	2131 (24.4%)
	>65	863 (9.9%)
**Gender**	Female	4962 (56.8%)
	Male	3781 (43.2%)
**Location**	Northern region	1957 (22.4%)
	Southern region	1756 (20.1%)
	Central Region	1482 (17.0%)
	Capital region	3548 (40.6%)
**Previous COVID-19 infection**	**Yes**	6189 (70.7%)
**Time of vaccinations**	Peak hour	2176 (24.9%)
	Regular time	6567 (75.1%)
**Vaccinated against seasonal Influenza virus**	**Yes**	1128 (12.9%)
**Type of vaccine**	BNT162b2	4511 (51.6%)
	BBIBP-CorV	2681 (30.7%)
	ChAdOx1 nCoV-19	1362 (15.6%)
	Sputnik V	189 (2.2%)

Pfizer and Sinopharm vaccines were the most frequently used

These VAEs were categorized into six types: timing (interval) error (69, 33.0%), dosing error (60, 28.7%), incorrect vaccine product (42, 20.1%), site/route error (17, 8.1%), documentation error (15, 7.2%), and other (6, 2.9%) (**Table 3**).

**Table 3:** Vaccines administration errors detected, their numbers (n) and percentage (%). (Total number of errors, N = 209).

Category	Scenario	Note	Number, n	Number (%)
Site/route	Incorrect site (i.e., site other than the deltoid muscle [preferred site] or anterolateral thigh [alternate site])		4^b^	1.9
	Incorrect route (e.g., subcutaneous)		13^b^	6.2
Intervals	Any COVID-19 vaccine dose administered prior to the recommended interval	• Error from personnel using immunization information system. • Vaccination reservation date not shown on Immunization information system.	15^b^	7.2
	Any COVID-19 vaccine dose administered after the recommended interval		44^b^	21.1
	Booster dose administered prior to the recommended interval		10^b^	4.8
Mixed series	Incorrect COVID-19 vaccine product inadvertently administered as part of 2-dose primary series or third booster dose.	Error from personnel using immunization information system. Vaccinee’s memory lapse.	42^a^	20.1
Diluent (Pfizer-BioNTech vial) Dosage (Sinopharm vial)	No diluent, resulting in higher than authorized dose (i.e., 0.3 ml of undiluted vaccine administered)		4^b^	1.9
	Vaccine is mixed with too little diluent, resulting in higher-than-authorized dose volume administered of the correct formulation. Higher-than-authorized dose volume administered of the correct formulation (pulling out more vaccine from Sinopharm vial).		23^b^	11.0
	• Vaccine is mixed with too much diluent, resulting in lower-than-authorized dose volume administered of the correct formulation. • Lower-than-authorized dose volume administered of the correct formulation (e.g., pulling out less vaccine from Sinopharm vial, leaked out, vaccinee pulled away). • Returning the remaining vaccine in a syringe (after administration) into the vaccine vial (Sinopharm).		33^b^	15.8
Not checking/documenting administration in the immunization information system.	Vaccine’s batch number entered incorrectly, leading to documenting wrong vaccine type. “Administered’ button not pressed on the electronic system after administering the first/second/third shot of the vaccine.	Error from personnel using immunization information system.	15^a^	7.2
Other	Wrong needle size, leading to severe shoulder pain/injury.		1^b^	0.5
	Recapping vaccine’s needle after administration causing injury to the vaccinator’s hand		1^b^	0.5
	Sterile procedure error (leaving vaccine syringe uncapped, not using gloves)		2^b^	1.0
	Not asking vaccinees about their allergy history, leading to anaphylactic shock.		2^b^	1.0

a: percentage of categories

b: percentage of subcategories

Of the 209 errors identified, 133 (63.6%) were deemed minor, 63 (30.1%) moderate, 12 (5.7%) potentially severe, and 1 (0.5%) life-threatening. Examples of VAEs and their severity are shown in Table 1 in S1 File.

Univariate analysis was done where results are shown in **Table 4**.

**Table 4:** Univariate analysis results.

Independent variable		Univariate OR (95%CI)	P value
**Age (years**)	16–18 vs >65	0.85 (0.55–0.97)	0.015
	18–35 vs >65	0.520 (0.11–0.87)	0.032
	35–65 vs >65	1.90 (0.235–2.40)	0.499
**Gender**	Female vs Male	1.31 (0.12–2.71)	0.137
**Location**	Northern region vs Capital region	0.48 (0.17–0.73)	0.015
	Southern region vs Capital region	2.25 (1.56–3.71)	0.001
	Central Region vs Capital region	0.39 (0.15–0.60)	0.031
**Previous COVID-19 infection**	Yes vs No	0.85 (0.65–3.50)	0.100
**Time of vaccinations**	Peak hour vs regular time	2.50 (1.70–4.51)	0.001
**Vaccinated against seasonal Influenza virus**	Yes vs No	0.54 (0.25–0.63)	0.041
**Type of vaccine**	BNT162b2 vs BBIBP-CorV	1.63 (0.72–1.69)	0.130
	ChAdOx1 nCoV-19 vs BBIBP-CorV	1.45 (0.58–4.70)	0.200
	Sputnik V vs BBIBP-CorV	2.03 (0.63–7.90)	0.381

Predictors for VAEs were those having received the vaccine in the Southern region compared to those in the Capital region (aOR: 1.92; 95%CI: 1.41–2.49; p = 0.001) and those who received the vaccine during peak hours compared to regular hours (aOR: 2.18; 95%CI: 1.58–3.86; p = 0.002) (**Table 5**).

**Table 5:** Predictors of VAEs.

Independent variable		AOR (95%CI)	P value
**Age (years**)	16–18 vs >65	0.74 (0.3–3.92)	0.156
	18–35 vs >65	0.82 (0.11–5.41)	0.351
**Location**	Northern region vs Capital region	0.68 (0.14–1.23)	0.091
	Southern region vs Capital region	**1.92 (1.41–2.49)**	**0.001**
	Central Region vs Capital region	1.26 (0.58–3.45)	0.086
**Time of vaccinations**	Peak hour vs regular time	**2.18 (1.5–3.86)**	**0.002**
**Vaccinated against seasonal Influenza virus**	Yes vs No	0.56 (0.32–1.09)	0.069

AOR: Adjusted odds ratio. CI: confidence interval. Bold AOR indicates **significant results.**

**Fig 1:**
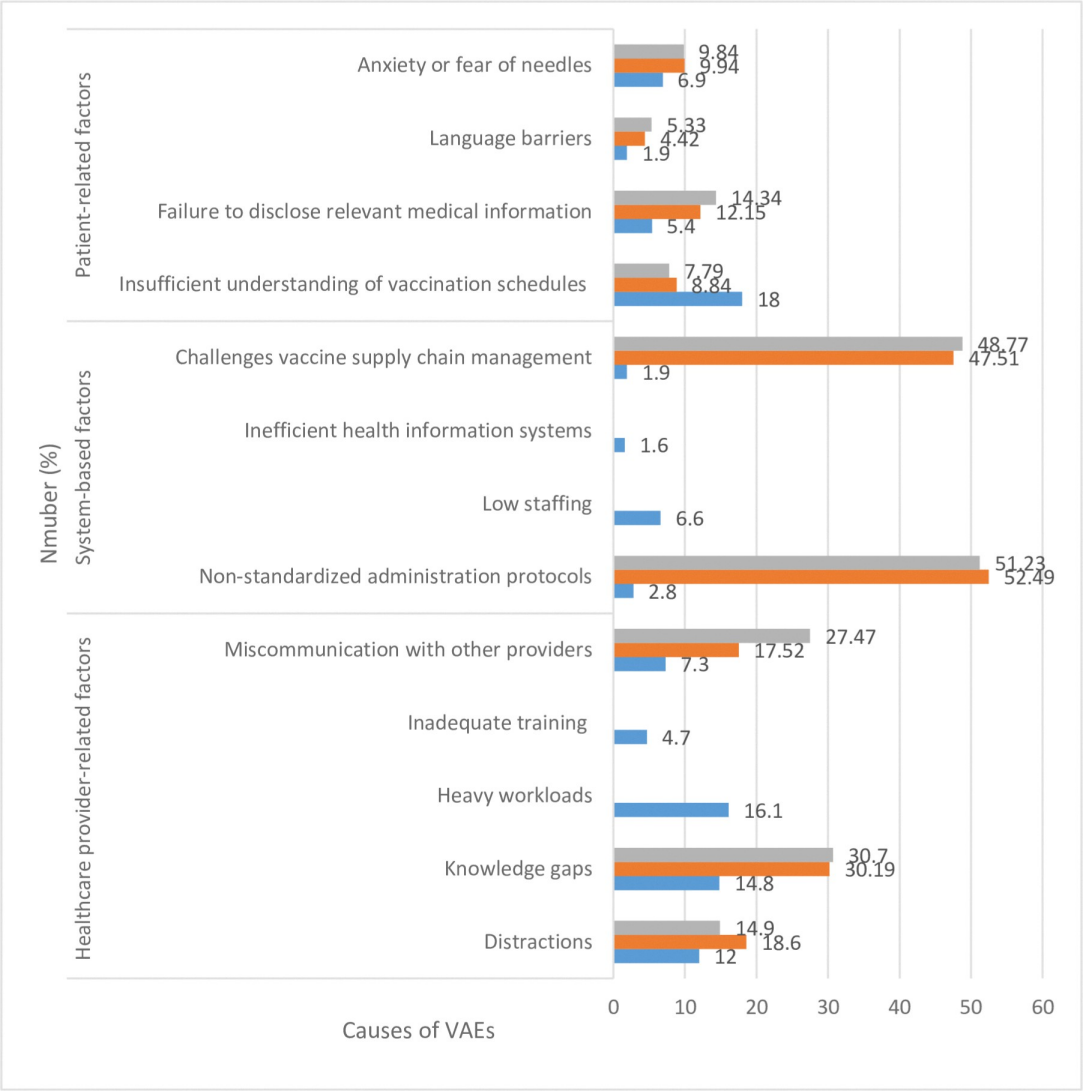
Potential causes of VAEs (N = 317).

Overall, there were three major contributing factors and 13 sub-factors to VAEs (**Fig 1**), which occurred 317 times during the study period. There were 174 (54.9%) healthcare provider-related factors, including heavy workload (16.1%, 51/317), knowledge gaps (14.8%, 47/317), and distractions (12.0%, 38/317). In addition, there were 102 (32.2%) patient-related factors, including insufficient understanding of vaccination schedules (18.0%, 57/317) and fear of needles (6.9%, 22/317).

## Discussion

This observational study, which is part of a national project investigating the safety and effectiveness of four types of COVID-19 vaccines [15, 20–26], prospectively investigated the prevalence, types, severity, predictors, and causes of COVID-19 vaccine administration errors (VAEs). Our multifaceted approach, implementing standardized procedure and combining a prospective point prevalence study with semi-structured interviews and voluntary error reporting, offered valuable insights into this critical aspect of the global vaccination effort. The study’s findings highlighted areas for improvement in vaccination practices, with implications for enhancing patient safety, optimising vaccine effectiveness, and bolstering public trust in immunization programmes.

Our study revealed a COVID-19 VAE prevalence of 2.4% in Jordan, a figure notably higher than the highest reported prevalence (1.15 per 10,000 doses) in the pre-pandemic era [16]. This discrepancy likely reflected the unprecedented scale and urgency of the COVID-19 vaccination campaign. The rapid development, approval, and rollout of multiple vaccines with varying protocols placed immense pressure on healthcare systems and providers [4]. This accelerated timeline likely contributed to increased occurrences of timing, dosage, and incorrect product errors, as healthcare workers grappled with swiftly adapting to new guidelines and unfamiliar vaccine-specific requirements.

This finding was consistent with other studies conducted during the pandemic, which also reported errors compared to pre-pandemic data. For instance, a study in Morocco found a concerning prevalence of VAEs related to COVID-19 vaccines (4 per million doses of vaccine administered), mainly attributed to the pressures of mass vaccination campaigns and the introduction of new vaccines [27]. This highlighted the need for robust training programmes specifically tailored to new vaccines and adapted to the challenges of mass vaccination campaigns.

The predominance of timing and dosage errors in our study further emphasized the need to address system-level factors that contribute to VAEs. These errors often stemmed from inadequate staffing, time pressures, and heavy workloads, particularly during peak vaccination hours (as evidenced by our findings), leading to hurried administration and reduced vigilance [18, 28]. This aligned with previous research highlighting the influence of workload on healthcare provider performance and its contribution to medication errors [29], which result in improper use of medicines, causing harm or even death [30, 31]. Implementing strategies to optimize staffing levels, streamline workflow processes, and ensure adequate workspace organization can mitigate these pressures and create an environment conducive to safe vaccination practices.

While the majority of errors identified were categorized as minor or moderate, the occurrence of potentially severe and even life-threatening errors, albeit less frequent, underscored the significant threat that VAEs posed to patient safety. Although intercepted by the covert observers in our study, these errors highlighted the potentially catastrophic consequences of VAEs. This reinforces the importance of vigilance and meticulousness, even when dealing with seemingly minor deviations from protocols. A study by the CDC analyzing VAEs data found that while rare, severe errors like administering the wrong vaccine type or incorrect route can have serious health consequences [17]. Efforts to minimize severe VAEs should focus on robust allergy screening protocols, clear communication of contraindications, and meticulous adherence to vaccine preparation and administration guidelines. For example, ensuring that vaccinators meticulously verify patient allergies and contraindications before administering a vaccine is paramount. Implementing standardized pre-vaccination checklists and leveraging technology, such as barcode scanning systems, can help reduce the risk of administering a vaccine to an individual with a known allergy.

Our study identified two significant predictors of VAEs: geographical location and timing of administration. The elevated risk observed in the Southern region compared to the Capital region likely reflected disparities in healthcare resources, training opportunities, and infrastructure across different geographical areas [32]. This finding emphasized the need for targeted interventions to address regional disparities and ensure equitable access to resources and training that support safe vaccination practices.

Furthermore, the increased likelihood of VAEs during peak vaccination hours reinforced the impact of workload and time constraints on healthcare provider performance. This underscores the importance of optimizing staffing levels during these periods, implementing strategies to manage patient flow effectively, and fostering a culture of safety that encourages reporting and learning from errors [8, 29]. This aligned with the WHO’s recommendations for ensuring safe injection practices, which emphasize the need for adequate staffing, workload management, and a supportive work environment [33].

Limitations of our study included the purposive sampling method, which may have limited the generalizability of the findings. The use of covert observers, while minimizing the Hawthorne effect [34], could have introduced observation bias. The small sample size of vaccinators precluded a robust analysis of their characteristics and their potential association with errors.

Despite its limitations, this study had several notable strengths. Firstly, its prospective design, involving real-time data collection during an active vaccination campaign, enhanced the accuracy of the findings and increased their relevance to real-world vaccination practices. Secondly, the multifaceted data collection approach, encompassing direct observation, semi-structured interviews, and voluntary error reporting, provided a comprehensive perspective on the nature and causes of VAEs. This triangulation of data strengthened the validity of the findings and offered richer insights than any single method could provide.

Furthermore, the study’s rigorous methodology, including the training of covert observers and the implementation of standardized procedures, strengthened confidence in the reliability and consistency of the data collected. The study’s setting within the context of a nationwide vaccination programme in Jordan provided valuable country-specific data and highlighted the importance of investigating VAEs within diverse healthcare contexts.

Our findings had important implications for improving vaccination programmes in Jordan. Prioritizing comprehensive and ongoing training for healthcare providers, particularly focusing on new vaccines and evolving administration guidelines, is crucial. Standardizing protocols, implementing technological solutions, such as barcode scanning and electronic health records, and fostering a culture of safety that encourages error reporting without fear of retribution can significantly reduce VAEs and improve vaccination outcomes.

Further research should focus on exploring the impact of interventions aimed at reducing VAEs, investigating the association between vaccinator characteristics and error rates with larger, more diverse samples, and examining the long-term consequences of VAEs on vaccine confidence and public health. By addressing the factors contributing to VAEs, we can ensure that vaccination programmes achieve their full potential in protecting individuals and communities from vaccine-preventable diseases.

## Conclusion

This study, the first to comprehensively examine COVID-19 vaccine administration errors (VAEs) in Jordan, reveals a concerning prevalence of 2.4%, exceeding rates reported in pre-pandemic vaccination efforts. Our findings underscored the significant challenges posed by the rapid rollout of multiple vaccines under unprecedented circumstances. The predominance of timing and dosage errors, particularly during peak hours, highlighted the detrimental impact of system-level pressures on healthcare provider performance.

Critically, the identification of potentially severe and even life-threatening errors, albeit less frequent, underscored the urgent need for targeted interventions. While these errors were mitigated through observer intervention in our study, they emphasized the potentially catastrophic consequences of VAEs and highlighted the need for rigorous safety protocols.

## Supporting information

S1 File (DOCX)
