# Supplementary material for: Investigating the point prevalence, types, severity, causes and predictors of vaccines administration errors during COVID-19 pandemic in Jordan
Source: PLoS One. 2025 Jan 3;20(1):e0312050. doi: 10.1371/journal.pone.0312050 (PMC12140077; doi:10.1371/journal.pone.0312050)
Supplement: S1 File — (DOCX) [file pone.0312050.s001.docx]

Supplementary material

**Table 1. Examples of the severity rating of vaccination administration errors (VAEs).**

| **Severity** | **Example** | **Impact** | **n (%)** |
| --- | --- | --- | --- |
| Minor | A vaccinator forgot to use a new alcohol swab to clean the injection site before administering a COVID-19 vaccine. | Minor breach of aseptic technique. The risk of infection is slightly elevated but likely minimal. | 133 (63.6%) |
|  | A vaccinator administered the vaccine slightly outside the deltoid muscle area, but still within the upper arm region. | Might cause slightly increased soreness or redness, but unlikely to affect vaccine effectiveness. |  |
|  | A vaccinator forgot to initial the vaccine information sticker on the patient's record. | Minor documentation error. Could cause confusion in tracking but easily rectified. |  |
| Moderate | Due to mixing with too little diluent during a busy vaccination drive, a patient received an overdosage of a COVID-19 vaccine. | The patient might experience increased pain and localized swelling at the injection site due to the larger volume. While vaccine efficacy is unlikely to be affected, the risk of systemic side effects (e.g., fever, fatigue) could be higher. | 63 (30.1%) |
|  | A patient scheduled for their second dose of a particular COVID-19 vaccine mistakenly received a different vaccine for their second dose due to a mix-up in the refrigerator. | Potential for reduced effectiveness of the vaccine series. May require additional doses or monitoring depending on the specific vaccines involved. |  |
|  | A vaccinator administered a vaccine intramuscularly, but the needle was shorter than recommended, resulting in the vaccine being injected too shallowly. | Potential for reduced vaccine effectiveness as the vaccine may not be deposited in the muscle tissue properly. |  |
| Severe | A vaccinator mistakenly could have administered a COVID-19 vaccine to a patient who was known to have severe allergy to flu vaccine. The patient's allergy information was documented on the database but overlooked during vaccine administration. | High risk of anaphylaxis, a life-threatening allergic reaction. | 12 (5.7%) |
|  | A multi-dose vial of COVID-19 vaccine was left out at room temperature for several hours instead of being returned to the refrigerator, potentially compromising its effectiveness. | Multiple patients might receive less effective or ineffective doses. Large-scale recall or revaccination may be necessary. |  |
| Life-threatening | A patient with a documented anaphylactic allergy to polyethylene glycol (PEG) contained in Pfizer vaccine could have accidentally been given that vaccine due to miscommunication during the screening process. | High likelihood of anaphylaxis, potentially leading to death if not treated immediately. | 1 (0.5%) |
